# Molecular Genetics of Primary Congenital Hypothyroidism: Established and Emerging Contributors to Thyroid Dysgenesis

**DOI:** 10.3390/ijms262210849

**Published:** 2025-11-08

**Authors:** Niki Dermitzaki, Anastasios Serbis, Maria Baltogianni, Dimitra Gialamprinou, Lida Eleni Giaprou, Chrysoula Kosmeri, Vasileios Giapros

**Affiliations:** 1 Neonatal Intensive Care Unit, School of Medicine, University of Ioannina, 45500 Ioannina, Greece; n.dermitzaki@uoi.gr (N.D.); mbalt@doctors.org.uk (M.B.); md07243@uoi.gr (L.E.G.); 2 Pediatric Department, School of Medicine, University of Ioannina, 45500 Ioannina, Greece; aserbis@uoi.gr (A.S.); chrisa.kosmeri@gmail.com (C.K.); 3 Second Neonatal Department and Neonatal Intensive Care Unit (NICU), “Papageorgiou” University Hospital, Aristotle University of Thessaloniki, 56403 Thessaloniki, Greece; gialamprinou@gmail.com

**Keywords:** congenital hypothyroidism, thyroid dysgenesis, molecular genetics

## Abstract

Congenital hypothyroidism (CH) is one of the most common endocrine disorders of childhood. The primary form of CH is attributable to thyroid dysgenesis (agenesis, hypoplasia, or ectopy) in 65–85% of cases, with the remaining cases being attributed to dyshormogenesis. Thyroid dysgenesis was considered a sporadic disease. However, the recent advantages of molecular techniques have significantly contributed to the understanding of the pathogenesis of the disease. The higher prevalence of congenital malformations and syndromes in patients with CH compared to the general population supports the genetic basis. This narrative review aims to provide an overview of the identified and potential genetic causes of thyroid dysgenesis. Mutations in ten genes involved in thyroid gland development during embryogenesis, *TSHR*, *PAX8*, *NKX2-1*, *NKX2-5*, *FOXE1*, *JAG1*, *NTN1*, *GLIS3*, *CDC8A*, and *TUBB1*, have been identified in cohorts of patients with thyroid dysgenesis. However, most cases remain unexplained. Novel candidate genes have been proposed. The extant evidence suggests that the pathogenesis of thyroid dysgenesis involves a spectrum of genetic etiologies, ranging from monogenic to multigenic, and that epigenetic or environmental factors may also contribute. As molecular techniques are continuously refined, future studies are expected to elucidate the complex genetic background of thyroid dysgenesis.

## 1. Introduction

Thyroid hormones are of critical importance for optimal development during early life. They are essential for growth, the maintenance of metabolic homeostasis, and the development and maturation of tissues and organs, including the central nervous system (CNS) [1,2]. Congenital hypothyroidism (CH) is characterized by a deficiency of thyroid hormones at birth [3]. With an estimated prevalence of 1 in 2000 to 3000 births, it represents the most prevalent congenital endocrine disorder in childhood. CH is one of the most common endocrine disorders in infants and children and the most common preventable cause of intellectual disability [4,5].

CH can be classified into three distinct categories, primary, secondary (central), and peripheral, depending on the origin of the defect [3,6]. Approximately 65–85% of cases of primary CH are attributed to thyroid dysgenesis, which involves thyroid aplasia, hypoplasia, or most commonly, ectopy [3,7]. The remaining 15–35% of primary CH is attributable to dyshormogenesis, a defect in the synthesis of thyroid hormones [3,6]. Nevertheless, several recent studies have reported rates of dyshormogenesis as high as 50%, likely attributable to regional variations, genetic factors, or the enhanced sensitivity of newborn screening [8,9]. Primary CH can be further classified into permanent and transient. Central CH is characterized by a deficiency in thyroid-stimulating hormone (TSH) production, which is most commonly associated with congenital hypopituitarism [3].

The thyroid gland is identified by the third or fourth week of gestation. It is formed from the thyroid diverticulum, a structural formation located in the floor of the primitive pharynx. This structure of endodermal origin gives rise to the follicular cells that produce thyroid hormones. It descends and fuses with the ultimobranchial bodies, which originate from the ventral recess of the fourth pharyngeal pouch, reaching its final position in front of the trachea at the base of the neck by the seventh week of gestation [10]. Parafollicular cells that produce calcitonin are generated from neural crest cells within the ultimobranchial bodies. The thyroid transcription factors NKX2-1, PAX8, FOXE1, and HEEX are crucial for the formation and migration of the thyroid gland during organogenesis and for maintaining a functional, differentiated state throughout life [11]. Following the processes of formation and migration, the ultimate phase is folliculogenesis, a differentiation process essential for hormonogenesis. Several genes are implicated in differentiation and hormonogenesis, including thyroglobulin (*TG*), TSH receptor (*TSHR*), thyroperoxidase (*TPO*), sodium/chloride symporter (*SLC5A5*), dual oxidase 2 (*DUOX2*), membrane transporters pendrin (*SLC26A4*), monocarboxylate transporter 8 (*SLC16A2*), and dehalogenase Dehall (*IYD*) [12]. The various types of CH are the consequence of deviations occurring at different stages of thyroid development, resulting in anatomical malformations or defects in thyroid hormone synthesis [1].

Thyroid dysgenesis is a group of disorders that arise from defects during the development of the thyroid gland. Poor survival or defects in the differentiation of the follicular cells result in partial or total absence of thyroid tissue, hypoplasia, and agenesis, respectively. The failure of thyroid precursors to migrate to their normal anatomic position leads to ectopy [13]. The most prevalent form of thyroid dysgenesis is ectopy, which accounts for 60–80% of cases. The most common location of the ectopic thyroid gland is the sublingual position. It is reported that thyroid agenesis is accountable for 20–30% of cases of thyroid dysgenesis, with the less common form, hypoplasia, accounting for approximately 5% [14]. Notably, in cases of thyroid hemiagenesis, the left lobe is absent in nearly 90% of affected individuals [15,16].

In cases of CH, early diagnosis and treatment are essential to avoid the potentially detrimental effects of insufficient thyroid hormones on neurodevelopment and growth [2]. Screening programs for CH have been implemented in many countries worldwide to ensure early diagnosis. As it is not possible to differentiate between permanent and transient CH during the neonatal period, prompt levothyroxine supplementation is necessary in all cases to prevent long-term consequences [6,17]. Thyroid agenesis and ectopy are typically associated with permanent CH. However, it has been demonstrated that patients with thyroid hypoplasia have frequently milder hormone disturbances and need lower doses of LT4 replacement. Indeed, cases of thyroid hypoplasia are associated with CH of variable severity, permanent or even transient [6,18,19]. Nevertheless, long-term follow-up is needed in all cases of CH to ensure adequate replacement therapy [4]. Research into the genetic background of CH cases is a promising and growing field of study. The primary objective of this research is to develop more accurate prognoses and individually tailored treatment and follow-up plans for patients.

In fewer than 5–10% of cases of thyroid dysgenesis, a genetic cause is identified. However, current evidence suggests that genetic factors are probably implicated in the pathogenesis of thyroid gland malformations [12,20,21,22,23]. The extant data from experimental animal and human studies suggest that the pathogenesis of thyroid dysgenesis likely involves a spectrum of genetic etiologies, ranging from monogenic to multigenic, and that epigenetic or environmental factors may also play a contributing role [20]. The rapid evolution of molecular studies in recent years, including next-generation sequencing (NGS) and whole-exome sequencing (WES), has provided an opportunity to identify new genes involved in thyroid dysgenesis, as well as to recognize the association between specific gene mutations, thyroid phenotype, and associated malformations [24]. The primary aim of this narrative review was to provide an overview of the current literature regarding the genes implicated in thyroid dysgenesis. A secondary objective was to summarize the malformations frequently associated with the implicated gene mutations to aid clinicians in performing targeted screening. In order to provide a comprehensive review of the extant literature, key studies were identified through targeted searching of relevant scientific online databases (PubMed, Google Scholar, Scopus) and by reviewing the reference lists of retrieved articles.

## 2. Genes Associated with Thyroid Dysgenesis (Table 1)

### 2.1. TSHR

The thyroid-stimulating hormone receptor (TSHR), a transmembrane receptor of follicular cells, is a member of the G-protein-coupled receptor (GPCR) family and is encoded by the *TSHR* gene, which is located on chromosome 14q31 and consists of 10 exons [25,26,27,28]. This seven-transmembrane receptor has been demonstrated to bind TSH with high affinity and regulate follicular cell differentiation and proliferation. Additionally, it is implicated in the synthesis of thyroid hormones [29].

The initial evidence for the role of TSHR in thyroid cell differentiation was identified in experimental animals. Stein et al. demonstrated that mice carrying loss-of-function mutations in the fourth transmembrane domain of the TSHR, leading to hyporesponsiveness of follicular cells to TSH, exhibited primary hypothyroidism and a hypoplastic thyroid gland with reduced follicular size, colloid, microvilli, and mitochondria [30]. Subsequent studies demonstrated that experimental animals carrying *TSHR* mutations presented with a normal thyroid gland size at birth, but following the first postnatal weeks, their thyroid glands were found to be hypoplastic [31,32]. These observations suggest that *THSR* is necessary for maintaining the volume and structure of the thyroid gland postnatally, rather than during embryogenesis [23]. Indeed, *TSHR* mutations have been demonstrated to be associated with thyroid gland hypoplasia, but not agenesis or ectopia [28].

In humans, the phenotypic spectrum of patients carrying *TSHR* loss-of-function mutations varies significantly, from euthyroid hyperthyrotropinemia to severe hypothyroidism with a hypoplastic thyroid gland, depending on the degree of TSH resistance [13]. The degree of TSH resistance is associated with the presence of heterozygous or homozygous mutations and the extent of functional impairment of the receptor [33]. Orthotopic thyroid hypoplasia and severe CH have been observed in recessively inherited homozygous or compound heterozygous loss-of-function mutations of the *TSHR* gene associated with severe TSH-resistance [28,34,35]. In contrast, autosomal dominant inherited heterozygous loss-of-function mutations are typically associated with less severe or subclinical CH and a normal-sized thyroid gland [13,33].

*TSHR* gene mutations have been identified as a prevalent underlying genetic factor associated with CH. A recent systematic review reported a prevalence of *TSHR* mutations ranging from 0% to 36% among different cohorts of patients with CH from various geographic regions [36]. Szinnai et al. reported a prevalence of 4.3% of homozygous and heterozygous *TSHR* mutations among patients with thyroid dysgenesis [14].

### 2.2. PAX8

Paired box transcription factor 8 (PAX8), a member of the mammalian Pax protein family, has a crucial role during embryogenesis. It is encoded by the *PAX8* gene, which is located on chromosome 2q12-q14 and consists of 12 exons. Binding to DNA sequences is achieved through a 128-amino-acid-long paired domain [13]. The expression of the PAX8 protein is initiated from the third week of embryonic development and continues throughout adulthood [37].

It has been demonstrated that PAX8 is imperative for thyroid cell development and specification during early embryonic period. Additionally, it is essential for maintaining the differentiated state and promoting proliferation at all stages of development [12]. Furthermore, PAX8 plays a role in controlling the transcription of thyroid genes, including thyroglobulin (*TG*), thyroperoxidase (*TPO*) and sodium/iodide symporter (*NIS*) [38]. In addition to its expression during thyroid development, experimental evidence has demonstrated its expression in the developing kidneys and myelencephalon of experimental animals [39].

Mansouri et al. demonstrated that *Pax8*-null mice presented with a hypoplastic thyroid gland, characterized by a deficiency of follicular cells. In the absence of thyroxine replacement, these mice exhibited impaired growth and died within two to three weeks. The development of the kidneys and CNS was found to be normal. The presence of thyroid hypoplasia was not observed in heterozygous *Pax8* mice [40].

In humans, in contrast to experimental animals, all affected patients are heterozygous for a loss-of-function mutation in the *PAX8* gene. The reported mutations most commonly involve exons 3–4, which encode the paired domain leading to a severe reduction in DNA-binding capacity [12,38]. *PAX8* mutations have been identified as a cause of CH and thyroid dysgenesis in both sporadic and familial cases [41]. In familial cases, autosomal dominant inheritance with incomplete penetrance and variable expressivity has been observed [12,42]. Thyroid hypoplasia is the most common anatomical malformation, but cases of agenesis and ectopy have also been reported. Furthermore, cases of both severe hypothyroidism and euthyroidism have been described in affected patients [41]. Significant variations in phenotype, regarding gland morphology and biochemical alterations, have been reported even in familial cases [43,44].

Although it represents one of the leading recognized genetic causes of thyroid dysgenesis, the overall reported prevalence of *PAX8* gene mutations in cohorts consisting of patients with thyroid dysgenesis is low. In cohorts from different geographic regions, *PAX8* mutations have been reported in 0.5–3.3% of patients with thyroid dysgenesis [43,44,45,46,47,48,49]. The association of *PAX8* mutations with urogenital malformations has also been reported [50,51,52,53].

### 2.3. FOXE1

The forkhead box E1 (*FOXE1*) or thyroid transcription factor 2 (*TTF2*) gene, which encodes a transcription factor (FOXE1) essential for thyroid gland development, is located on chromosome 9q22 and contains a single exon [13,54]. During embryogenesis, *FOXE1* has been detected to be expressed in the thyroid primordium, pharyngeal, esophageal, tracheal, and thymus epithelium [55].

De Felice et al. observed that *Foxe1*-null mice presented with cleft palate and either an ectopic or absent thyroid gland [56]. In human subjects, homozygous loss-of-function mutations in the *FOXE1* gene have been associated with severe hypoplasia or agenesis of the thyroid gland, as part of as the so-called Bamforth–Lazarus syndrome [14]. In addition to thyroid dysgenesis, patients present with cleft palate and spiky hair that may be accompanied by bifid epiglottis and choanal atresia [57]. Currently, eight pathogenic variants of *FOXE1* have been associated with Bamforth–Lazarus syndrome [57]. There have been no reported cases of isolated thyroid dysgenesis, and thus screening for *FOXE1* mutation in patients with dysgenesis should be guided by the presence of associated features [14].

### 2.4. NKX2.1

The *NKX2.1* gene, located on chromosome 14q13 and comprising three exons, encodes a homeodomain-containing protein, NKX2.1, also known as thyroid transcription factor 1 (TTF-1) [58]. During development, it is expressed in the thyroid gland, the lungs, and regions of the CNS, and its expression is maintained in follicular cells, hypothalamus, and pneumocytes (type II) throughout adulthood [55].

More specifically, it has been demonstrated that NKX2.1 plays a crucial role in the survival of follicular cell precursors, folliculogenesis, and differentiation during early embryogenesis, as well as in the regulation of the expression of several thyroid-specific genes, including *TG*, *TPO*, and *NIS* [59,60]. In the lungs, *NKX2.1* regulates the expression of several genes, including those encoding surfactant proteins A, B, and C [61,62].

Kimura et al. demonstrated that homozygosity for mutations in the *Nkx2.1* gene led to lethality in mice, due to the absence of Nkx2.1 expression. This was characterized by a lack of lung parenchyma, agenesis of the thyroid gland, and extensive defects in the forebrain. Conversely, mice carrying heterozygous mutations exhibited normal development [60].

Conversely, in human subjects, heterozygous mutations have been observed in affected patients, thus suggesting an autosomal dominant mode of inheritance. De novo mutations are frequently reported [23]. Haploinsufficiency of the *NKX2.1* gene has been associated with a distinct complex phenotype known as the brain-lung-thyroid (BLT) syndrome [63]. The BLT syndrome is characterized by different combinations of neurological implications such as hypotonia during infancy evolving to benign hereditary chorea, lung morbidities such as respiratory distress syndrome at birth evolving to interstitial lung disease, and CH [64]. The extent of thyroid abnormalities and the severity of CH vary significantly among affected individuals, ranging from hypethyrotropinemia (61%) to overt hypothyroidism (39%) [65]. Most affected cases present with an orthotopic normal-sized thyroid gland, followed by hypoplasia or hemiagenesis (35–50%) and agenesis (10%) [64,65,66]. Thyroid ectopy has been reported only in isolated cases [64].

A considerable degree of phenotypic variability in terms of the systems involved is observed in patients with BLT. A recent systematic review of 46 studies involving 113 patients with *NKX2.1*-related disorders reported that 57.8% of subjects exhibited the full triad of symptoms [67]. CNS is the most commonly affected system (93%) followed by thyroid abnormalities (87%) and respiratory system (54%) [67].

The presence of *NKX2.1* mutations has not been identified in cohorts of non-syndromic thyroid genesis [14,23]. However, in the clinical context of suspected BLT syndrome, genetic testing for *NKX2.1* mutations is recommended [4].

### 2.5. NKX2.5

*NKX2.5*, a homeodomain-containing transcription factor, belongs to the same homeobox gene family as *NKX2.1*. NKX2.5 is encoded by the *NKX2.5* gene, which is located on chromosome 5q35 and comprises two exons [68,69]. It has been demonstrated that NKX2.5 has a pivotal role in the embryonic development of the heart, and mutations have been linked with congenital heart disease [70,71].

Several studies have investigated the role of NKX2.5 in thyroid development and the pathogenesis of CH and thyroid dysgenesis [68,69]. Denice et al. demonstrated that *Nkx2.5* null mouse embryos presented with thyroid bud hypoplasia, suggesting that Nkx2.5 has a role in thyroid early development. Furthermore, a total of four patients with three heterozygous missense changes in *NKX2.5* were identified in a cohort of 241 cases of thyroid dysgenesis [72]. Following the findings of this study, it was hypothesized that *NKX2.5* mutations could be a causative factor in thyroid dysgenesis. A few cases of patients with thyroid dysgenesis and heterozygous mutations of the *NKX2.5* have been described [73,74,75,76]. However, further research is needed to establish the precise role of this gene in the pathogenesis of thyroid gland developmental abnormalities. It has been suggested that other genes or factors may modulate the expression and penetrance [77]. Indeed, several studies in CH cohorts from various geographic regions revealed no pathogenic mutations of the *NKX2.5* gene associated with thyroid dysgenesis [22,78,79,80]. Therefore, the contribution of *NKX2.5* mutations to CH remains equivocal, and it is not currently regarded as a significant contributor to thyroid dysgenesis [81].

### 2.6. GLIS3

*GLIS3*, a member of the GLI-similar zinc finger protein family, is located on chromosome 9p24. It encodes a nuclear protein comprising five zinc finger domains, which serves as a downstream regulator of the Sonic Hedgehog pathway (SHH). Given its involvement in both the activation and repression of transcription during the early stages of development, gene mutations are potentially associated with multisystem involvement [82]. The interaction of GLIS transcription factors with specific tissue regulatory genes during embryonic life is responsible for the manifestation of a variety of disorders, including neonatal diabetes, CH, polycystic kidney disease, hepatic fibrosis, facial dysmorphism, glaucoma, osteopenia, and skeletal abnormalities [83,84,85,86].

Recessively inherited biallelic loss-of-function mutations are associated with permanent neonatal diabetes, CH, and, in many cases, involvement of various organs. Heterozygous or homozygous single-nucleotide polymorphisms are associated with thyroid disorders [87,88,89]. Thyroid disorders are a consistent finding among affected patients. However, a considerable degree of variation in thyroid gland morphology and the severity of CH is observed, ranging from agenesis to reduced colloid with interstitial fibrosis to normal gross anatomy and echotexture of the thyroid gland. A distinct manifestation of the disease in patients with an apparently normal thyroid gland is a temporary dysregulation of TSH while on LT4 treatment, which has been reported to improve by dividing the daily dose of LT4 into three or four doses [82,90].

Conclusively, the presentation of permanent neonatal diabetes and CH is indicative of *GLIS3* gene mutations. In such cases, the performance of genetic testing and a detailed examination of other organ systems is recommended.

### 2.7. JAG1

The *JAG1* gene, which is located on chromosome 20p12.2, encodes the Jagged1 protein, a transmembrane protein that acts as a ligand for Notch signaling pathway activation [91]. The Notch pathway is an evolutionarily conserved signaling system for cell-to-cell communication, essential for maintaining the homeostasis, development, and specification of many tissues [92]. As demonstrated in in vivo studies, Notch signaling is essential for thyroid development [93]. Porazzi et al. observed that interruption of the Notch pathway in *Jag1* mutant zebrafish resulted in thyroid hypoplasia and impaired thyroxine production [94].

In humans, heterozygous loss-of-function mutations of *JAG1* are associated with Alagille syndrome, which is characterized by multisystem involvement that includes the heart, liver, kidneys, skeleton, eyes, and distinctive facial features [95]. De Filippis et al. evaluated the thyroid function in a cohort of 21 patients with Alagille syndrome. Six patients were diagnosed with non-autoimmune hypothyroidism, of whom two had thyroid hypoplasia. Moreover, among 100 unrelated patients with CH, they reported two heterozygous *JAG1* variants in four patients (three with thyroid dysgenesis) [96]. In a recent study, Li et al. analyzed *JAG1* in a cohort of 381 patients with CH. They reported ten likely pathogenic missense variants of *JAG1* in 25 patients (3.08%). Among the eight patients with thyroid dysgenesis, seven carried a single *JAG1* variant, suggesting that *JAG1* variants primarily cause thyroid dysgenesis through a monogenic model [91].

Therefore, current data support the role of *JAG1* mutations in the pathogenesis of thyroid dysgenesis, justifying the examination of these mutations in molecular studies.

### 2.8. NTN1

The *NTN1* gene encodes Netrin-1, which is a member of the Netrin family of extracellular proteins that regulate cell migration and survival during early development and beyond. Netrin-1 is involved in several developmental processes, including synaptogenesis and axon guidance, angiogenesis, cell migration, and apoptosis [97].

The role of *NTN1* mutations in thyroid dysgenesis remains to be established. In a cohort of 161 unrelated patients with thyroid dysgenesis, one patient with thyroid ectopia and a ventral septal defect was diagnosed with *NTN1* deletion. Two paralogous homologs of the human *NTN1* gene are expressed in zebrafish, namely *ntn1a* and *ntn1b*. Opitz et al. demonstrated that neither of these is expressed in zebrafish thyroid tissue. The expression of *ntn1a* is observed in the pharyngeal arch, and deficient embryos are characterized by thyroid hypoplasia in addition to structural heart defects. The authors suggest that thyroid hypoplasia in affected embryos resulted from the dysplastic vasculature, rather than a direct thyroid defect [98]. However, additional evidence is needed to determine the role of *NTN1* mutations in thyroid dysgenesis.

### 2.9. CDCA8

Borealin, encoded by the *CDCA8* gene located on chromosome 1, is a key constituent of the chromosomal passenger complex (CPC). The CPC has a critical functional role in chromosome segregation and cytokinesis [99].

Current evidence supports the role of borealin thyroid gland morphogenesis and function [100,101]. Borealin-deficient mice have been shown to present with altered thyroid development at birth, and goiter, disorganized follicles, and additional structural abnormalities at four and 18 months, respectively [101]. Carre et al. demonstrated that borealin is expressed in humans during the early stages of development, at 8 and 12 weeks of gestation. The role in thyrocyte migration and adhesion was documented. Low expression was observed in adult thyroid tissue [100].

To date, four mutations in the *CDCA8* gene have been identified. These all affect the region of the gene responsible for coding one segment of the protein, which is critical for the processes of thyrocyte adhesion and migration [24]. In a cohort of patients with thyroid dysgenesis, homozygous *CDCA8* mutations were identified in two cases within a blood-related family and heterozygous in two other sporadic cases. Different types of thyroid anatomical variations were observed, including ectopy, agenesis, and hemiagenesis [100]. Zhou et al. reported a novel *CDCA8* mutation in a heterozygous state in a cohort of 25 patients with thyroid dysgenesis, who were studied using next-generation exome sequencing [102].

### 2.10. TUBB1

*TUBB1* (Tubulin, Beta 1, class VI) is located on chromosome 20q13.32 and encodes a 451 amino acid protein, β-tubulin. The interaction between β-tubulin and α-tubulin is critical for the structure of microtubules, which are integral components of the eukaryotic cytoskeleton [103].

Stoupa et al. documented that *TUBB1* is expressed in the thyroid tissue of both humans and mice during early development and adulthood. *Tubb1*-knockout mice exhibited defects in thyrocyte proliferation, migration, impaired thyroid hormone production, and large platelets. The authors identified three novel mutations in the *TUBB1* gene, in homozygous or heterozygous state, in patients with thyroid dysgenesis and large platelets. Thyroid ectopia was the most prevalent thyroid defect, with hypoplasia and thyroid gland asymmetry also being reported [104]. In a recent study, Wang et al. reported the identification of four patients with a heterozygous pathogenic variant of the *TUBB1* gene in a cohort of 289 patients with dysgenesis [105].

**Table 1:** Overview of the genes associated with thyroid dysgenesis.

Gene	Location	Mode of Inheritance	Gene Product	Evidence	Type of Dysgenesis	Associated Malformations
*TSHR* [13,25,106]	14q31	AD, AR	TSHR	Established	Hypoplasia	-
*PAX8* [20,42,50]	2q12-q14	AD	PAX8	Established	Hypoplasia (most common), agenesis, ectopy	Urogenital malformations
*FOXE1* [14,54]	9q22	AR	FOXE1 (TTF2)	Established	Hypoplasia, agenesis	Bamforth–Lazarus syndrome: cleft palate, spiky hair, bifid epiglottis, choanal atresia
*NKX2.1* [23,58,64]	14q13	AD	NKX2.1 (TTF1)	Established	Hypoplasia, agenesis	Brain-lung-thyroid syndrome (hypotonia at birth, benign hereditary chorea, RDS at birth, interstitial lung disease)
*NKX2.5* [68,70,81]	5q35	AD	NKX2.5	Candidate	Uknown role	Cardiac defects
*GLIS3* [82,83,87]	9p24	AR	GLI-zinc finger protein family 3	Established	Hypoplasia, agenesis	Permanent neonatal diabetes, polycystic kidney disease, hepatic fibrosis, facial dysmorphism, glaucoma, osteopenia, skeletal abnormalities
*JAG1* [91,96]	20p12.2	AD	Jagged-1	Emerging	Hypoplasia	Alagille syndrome: cardiac, liver, kidneys, skeleton, eye defects, facial dysmorphism
*NTN1* [97,98]	17p13.1	AD	Netrin-1	Candidate	Ectopia	Cardiac defects, arthrogryposis, congenital mirror movement disorder
*CDCA8* [24,99,102]	1p34.3	AD, AR	Borealin	Established	Ectopia, agenesis	-
*TUBB1* [103,104]	20q13.32	AD, AR	β1-tubulin	Emerging	Ectopia, hypoplasia	Large platelets

*TSHR*: thyroid-stimulating hormone receptor; *PAX8*: Paired box transcription factor 8; *NKX2.1*: NK2 Homeobox 1; *NKX2.5*: NK2 Homeobox 5; *FOXE1*: forkhead box E1; *TUBB1*: Tubulin, Beta 1, class VI; TTF2: thyroid transcription factor 2; TTF1: thyroid transcription factor 1; AD: autosomal dominant; AR: autosomal recessive.

### 2.11. Novel Candidate Genes

Rapid advances in genetic studies have revealed new candidate genes that may be involved in thyroid dysgenesis. *TRPC4AP* variants have been identified in a cohort of patients with thyroid dysgenesis. Thyroid hypoplasia was observed following the knockdown of *Trpc4ap* in *Xenopus laevis* [107]. Recently, Sun et al. examined 98 patients with CH who did not carry known gene mutations using WES. They identified a patient with biallelic variants of the eukaryotic translation initiation factor 4B (EIF4B) gene and thyroid hypoplasia. Knockdown of *Eif4b* in zebrafish has been associated with thyroid dysgenesis [108]. Another gene potentially implicated in thyroid dysgenesis is the *ZBTB26* gene. Following the identification of *ZBTB26* variants in a cohort of patients with thyroid dysgenesis, Vick et al. studied the effect of *Zbtb26* knockdown in *Xenopus laevis* and demonstrated hypoplastic thyroid anlagen [109]. Similarly, *GBP1* has been proposed as a candidate gene associated with thyroid dysgenesis. Yang et al. identified three CH patients, two of whom had thyroid dysgenesis, with heterozygous variants of *GBP1*. The role of *Gbp1* in thyroid morphogenesis has been demonstrated in zebrafish [110]. In addition to the identification of novel candidate genes, the potential role of genes known to be involved in thyroid dyshormogenesis, including *SLC264A*, *DUOX2*, and *TPO*, has also been suggested [111,112]. However, the role of these genes, both known and novel, in the pathogenesis of thyroid dysgenesis remains uncertain, and further studies are needed to provide conclusive evidence.

## 3. Congenital Malformations and Syndromes Associated with CH

CH is associated with a high prevalence of concomitant congenital malformations (Table 2). The high rates of malformations and associated syndromes suggest that CH is probably not an isolated defect, but rather a part of a developmental sequence arising early during embryogenesis [113]. According to the results of a national population-based registry Finnish study, the rate of congenital malformation in patients with CH was 15.1% and 7.4% in controls (*p* < 0.001), and the rate of multiple malformations was 3.2% and 0.4% (*p* < 0.001), respectively [114]. This is in accordance with the results of several studies reporting significantly higher rates of congenital malformations in patients with CH [115,116].

The higher prevalence of congenital malformations in patients with CH compared to the general population supports the genetic basis of the disease [103,108]. It has been suggested that genetic factors during the initial phases of morphogenesis may potentially interfere with the development of various organs, thereby resulting in different organ involvement [112,117,118]. Indeed, several genes associated with thyroid dysgenesis, including the *NKX2.1*, *NKX2.5*, *PAX8*, *GLIS3*, *FOXE1*, *JAG1*, and *NNT1*, are implicated in the embryogenesis of other organs (Table 1) [119].

The development of the thyroid gland and the heart is closely related during the initial phase of embryonic life [114]. Genes involved in thyroid embryogenesis, such as *NKX2.5* and the homeobox gene *HEX*, have been associated with cardiac malformations [113]. Cardiac defects, mainly atrial septal defect, patent ductus arteriosus, and ventricular septal defect, are the malformations most commonly associated with CH, ranging from 5.55 to 29% in different cohorts (Table 2) [119]. The urogenital, musculoskeletal, and gastrointestinal systems are also amongst the systems most commonly affected [114].

Monroy-Santoyo et al. reported an overall prevalence of congenital malformation of 24% in patients with CH. The authors reported a higher prevalence of concomitant congenital anomalies in the group of patients with thyroid agenesis. Conversely, patients with ectopia demonstrated the lowest incidence of malformations [115]. This finding is consistent with other studies suggesting that thyroid ectopia is most commonly associated with isolated CH [120,121,122]. Tuli et al. also reported the highest rate of congenital malformation in patients with agenesis (53.3%) and the lowest in patients with ectopia (12%) [122]. It has been suggested that this may be attributable to specific genes implicated in the pathogenesis of agenesis, which may play a role in the development of other organs.

In addition to the high rates of associated malformations, thyroid dysgenesis may be part of a syndrome. One of the most common syndromes associated with thyroid dysgenesis is Bamforth–Lazarus syndrome. This is caused by mutations in the *FOXE1* gene and is characterized by thyroid dysgenesis, cleft palate, spiky hair, and, in some cases, bifid epiglottis or choanal atresia [57]. Mutations in the *NKX2.1* gene have been associated with brain-lung-thyroid syndrome, which is characterized by different combinations of neurological and lung morbidities, and CH [59]. Alagille syndrome, characterized by multisystem involvement affecting the heart, liver, kidneys, skeleton, and eyes, has been associated with heterozygous loss-of-function mutations of *JAG1*. Evidence has demonstrated the implication of these mutations in thyroid dysgenesis [91,95,96]. The T-box transcription factor (*TXB1*), which has been linked to the 22q deletion syndrome phenotype, has been shown to regulate thyroid embryonic development [123,124]. Thyroid hypoplasia or agenesis is one of the possible anomalies of the blepharocheilodontic syndrome, a condition characterized by eyelid malformations, cleft lip/palate, and dental anomalies. This syndrome has been associated with heterozygous mutations in the *CDH1* and *CTNND1* genes [125]. Individuals with Down syndrome have been shown to have a 35 times higher risk of CH of various severity when compared to the general population [126]. Studies on experimental animals have demonstrated that the *DYRK1A* gene, which is located in the critical region of chromosome 21 associated with Down syndrome, is probably implicated in cases of thyroid dysgenesis [24,127,128].

CH has been demonstrated to be associated with the presence of anatomical malformations, with reported coexistence rates ranging from 8 to 59% of cases [113,114,115,116,117,118,119,128,129,130,131]. The extant evidence indicates that genetic factors may be implicated in the pathogenesis of extrathyroidal malformations in patients with CH. The recognition of specific mutations could facilitate the implementation of targeted screening procedures for the early detection of concomitant malformations, thus enabling appropriate management strategies.

**Table 2:** Studies evaluating the presence of associated malformation in patients with congenital hypothyroidism.

Author	Population	Congenital Malformations
Olivieri, 2002 [129]	1420	Cardiovascular 5.5% Urogenital 0.4% Musculoskeletal 1% Gastrointestinal 0.5% Nervous system 0.8%
Kreisner, 2005 [130]	76	Cardiovascular 10.5%
Gu, 2009 [116]	1520	Cardiovascular 8.9% Urogenital 1.58% Musculoskeletal % Gastrointestinal 2.41% Nervous system 1.05%
Kumar, 2009 [131]	980	Cardiovascular 17.6% Urogenital 8.9% Musculoskeletal 1.2% Gastrointestinal 2.9%
Reddy, 2010 [113]	17	Cardiovascular 29% Neural tube defects 41%
Monroy-Santoyo, 2011 [115]	212	
Ravazi, 2012 [117]	150	Cardiovascular 4.9% Developmental hip dysplasia 2.6%
Wędrychowicz, 2019 [119]	54	Cardiovascular 18% Urogenital 9.25% Musculoskeletal 11.1% Gastrointestinal 12.96% Respiratory 9.25% CNS 5.55%
Tuli, 2020 [122]	105	Cardiovascular 16.1% Urogenital 6.7% Musculoskeletal 4.8% Gastrointestinal: 4.8%
Mazahir, 2020 [118]	204	Cardiovascular 16% Urogenital 14%
Danner, 2023 [114]	438	Cardiovascular 6.4% Urogenital 1.4% Musculoskeletal 5.3% Facial, cleft lift/palate:1.4%

## 4. Discussion

Thyroid dysgenesis was formerly regarded as a sporadic disorder. Extensive research and advances in molecular techniques have led to the recognition that the pathogenesis of thyroid dysgenesis involves a spectrum of genetic etiologies [13,14].

Nevertheless, several factors argue against monogenic germline mutations and Mendelian inheritance as the predominant cause of thyroid dysgenesis [132]. The occurrence of thyroid dysgenesis is primarily non-syndromic and sporadic, with 98% of cases being non-familial, and a high discordance rate of 92% reported in monozygotic twins [133]. It also presents a sexual predilection, with a female-to-male ratio of approximately 3:1 [119,120]. However, genetic predisposition is supported by the observation of ethnic differences, with a high incidence in Hispanics and Caucasians and rarity in African ancestry [13,21]. Moreover, it has been demonstrated that first-degree relatives of patients with thyroid dysgenesis are 40 times at risk of thyroid developmental abnormalities than the general population [134,135].

However, mutations in one of the causative genes, *TSHR*, *PAX8*, *NKX2-1*, *NKX2-5*, *FOXE1*, *JAG1*, *NTN1*, *GLIS3*, *BOREALIN*, *TUBB1* are identified in less than 5% of cases [24]. This indicates the potential involvement of additional, as yet unidentified, genetic or non-genetic mechanisms in the pathogenesis of thyroid dysgenesis.

The frequent sporadic occurrence and the high phenotypic variability and penetration observed among family members in terms of thyroid anatomy and hormonal profile led to the hypothesis that oligogenicity might contribute to the pathogenesis of CH [7]. Amendola et al. were the first to demonstrate the multigenic origin of the disease in mice. The authors observed that compound heterozygous mutations of the *Tift1* and *Pax8* genes resulted in thyroid hypoplasia. Single heterozygous mutations of either of these genes did not result in thyroid dysgenesis [136]. The oligogenic involvement in the pathogenesis of CH in humans has been demonstrated using NGS techniques, which enable the parallel analysis of a variety of genes [137]. De Filippis et al. reported oligogenic involvement in 22% of a cohort of 177 patients with CH, 83 of whom had thyroid dysgenesis. In contrast, recessive monogenic forms were present in less than 3% of patients. It was evident that the combination of rare variants, which would be associated with modest or negligible impairment when expressed alone, can be implicated in CH pathogenesis. These combinations may comprise variants that act at different levels of thyroid morphogenesis, and/or hormonogenesis [138].

In support of the multifactorial nature of thyroid dysgenesis, polymorphisms in the polyalanine tract of the *FOXE1* gene have been demonstrated to modulate the genetic susceptibility for thyroid dysgenesis. The presence of 16 alanines, either in a heterozygous (14/16) or homozygous (16/16) state, has been shown to act as a protective factor for thyroid dysgenesis, in contrast to the most common variant (14/14) [139].

It has been suggested that non-Mendelian mechanisms of inheritance may be implicated [140]. The two-hit hypothesis, suggesting that epigenetic factors or somatic mutations at an early stage of development may lead to a loss of function in a gene involved in thyroid gland development, has been proposed to explain the sporadic occurrence and observed discordance between monozygotic twins [140]. However, to date, there is an absence of definitive evidence that could substantiate this hypothesis [24].

## 5. Conclusions

The understanding of the genetic basis of thyroid dysgenesis has expanded considerably in recent years, mainly due to rapid advancements in molecular techniques. Despite the significant progress that has been made, considerable gaps remain in our understanding of the pathogenesis of thyroid dysgenesis, and the etiology of the majority of cases remains unexplained. Evidence suggests considerable genetic heterogeneity and variable expressivity. Genes that have been identified as critical during thyroid embryogenesis, such as *PAX-8*, *NKX2.1*, *NKX2.5*, *GLIS3*, and *FOXE1*, have been detected as causative factors in a minor proportion of cases. Novel genes have been identified and suggested as potential contributors to thyroid dysgenesis, with future studies expected to provide more robust evidence. Future detection of causative variants that have not yet been identified, in conjunction with further research into oligogenic involvement, epigenetic and environmental factors, has the potential to enhance our understanding and improve the diagnostic capability. Identification of the genetic etiology on an individual basis will facilitate a tailored approach in patients with thyroid dysgenesis, targeted screening, and enhanced outcomes.

## Data Availability

No new data were created or analyzed in this study.
